# The influence of venous tumor thrombus combined with bland thrombus on the surgical treatment and prognosis of renal cell carcinoma patients

**DOI:** 10.1002/cam4.3264

**Published:** 2020-07-06

**Authors:** Zhuo Liu, Li Zhang, Peng Hong, Liwei Li, Shiying Tang, Xun Zhao, Qiming Zhang, Guodong Zhu, Ran Peng, Binshuai Wang, Zhigang Chen, Zhenghui Sun, Feilong Yang, Guoliang Wang, Xiaojun Tian, Shudong Zhang, Yi Huang, Hongxian Zhang, Cheng Liu, Shumin Wang, Lulin Ma

**Affiliations:** ^1^ Department of Urology Peking University Third Hospital Beijing China; ^2^ Department of Ultrasound Peking University Third Hospital Beijing China; ^3^ Department of Radiation Oncology Peking University Third Hospital Beijing China

**Keywords:** bland thrombus, prognosis, renal cell carcinoma, surgical treatment

## Abstract

**Objective:**

To describe the clinical characteristics of renal cell carcinoma (RCC) with venous tumor thrombus (VTT) and bland thrombus (BT), and to evaluate the influence of BT on surgical treatment and cancer‐specific survival (CSS) of RCC with VTT.

**Methods:**

We retrospectively reviewed clinical data of 123 patients with RCC and VTT, who underwent surgical treatment in our center between February 2015 and May 2018. Patients were divided into the BT group (21 patients) and non‐BT group (102 patients). Chi‐square and Mann‐Whitney *U* test were used for categorical and continuous variables respectively. Univariable log‐rank tests and multivariable Cox regressions were conducted to evaluate the prognostic significance of each variable. Kaplan‐Meier plots were performed to evaluate the influence of BT.

**Results:**

In the delayed phase of enhanced magnetic resonance imaging (MRI), BT and VTT had difference. Patients were divided according to the relative position of BT: proximal end BT (one patients), contralateral renal vein BT (two patients), distal end BT (12 patients), and multiple BT (six patients). The average length of BT was 8.4 ± 5.8 cm (range: 0.6‐20.0 cm). Patients with BT had longer operative time (*P* = .001), more surgical blood loss (*P* = .004), higher proportion of open surgery (*P* = .006), more postoperative complications (*P* = .011). BT (hazard ratio [HR] = 3.323, *P* = .007) were independent risk factors for poor prognosis.

**Conclusions:**

In the delayed phase of enhanced MRI, BT showed no obvious enhancement, while VTT usually showed enhancement. This was an important basis for preoperative imaging diagnosis of BT. The presence of BT increases the difficulty of surgery, and is correlated with adverse survival outcomes in patients with RCC and VTT.

## Graphical abstract

MRI is an important examination for RCC patients with VTT and BT. BT has adverse influence on surgical treatment and survival.

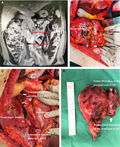

## 1 INTRODUCTION

Renal cell carcinoma (RCC) is a common malignant tumor in the urinary system.^1^ 4%‐10% of cases of local advanced RCC are concomitant with inferior vena cava (IVC) tumor thrombus (TT).^2^, ^3^, ^4^ For some cases of RCC with concomitant venous tumor thrombus (VTT), bland thrombus (BT) may be present at the same time. BT is a nontumor thrombus, mainly composed of activated platelets, macrophages, and fibrin.^5^ Its formation mechanism is unclear and may be related to stasis or hypercoagulability of venous blood.^6^ After the tumor cells enter the blood, they may activate the platelets. The activated platelets combine with the tumor cells forming TTs, which can enhance the ability of tumor cells to adhere to the vascular endothelial cells and protect the tumor cells from blood flow.^7^, ^8^, ^9^ The presence of BT increases the difficulty of diagnosis and surgical treatment for patients with RCC and VTT.^10^, ^11^ It is reported that BT is associated with adverse survival outcomes in patients with RCC and VTT. In multiple‐matching, propensity‐score‐matched cohorts, the presence of BT was associated with lower median cancer‐specific survival (CSS) (28.1 vs 76.7 months, *P* < .001).^2^

In this study, we present the clinical characteristics of RCC with VTT and BT, and the influence of BT on the surgery and CSS for RCC with VTT.

## 2 PATIENTS AND METHODS

### 2.1 Patients

We retrospectively reviewed the clinical data of 153 patients with RCC and VTT between February 2015 and May 2018. One hundred and twenty‐three patients, who underwent surgical treatment and were diagnosed pathologically with RCC, were included in this study. The inclusion criteria were as follows: (a) imaging examination showed that renal tumor was accompanied by VTT; (b) patients underwent radical nephrectomy and IVC thrombectomy; and (c) postoperative pathological type was RCC. The exclusion criteria were as follows: (a) patients with recurrent TT after radical nephrectomy who only underwent IVC thrombectomy; and (b) patients who were lost to follow‐up. The procedure for patient selection is shown in the Figure S1. None of the enrolled patients were treated with targeted drugs before surgery. Among 123 patients enrolled, 21 patients had concomitant BT and 102 had no BT. In the 21 patients with BT, there were 16 men and five women. Thirteen renal tumors were located on the right side and eight on the left. According to Mayo classification,^2^ one patient had level I VTT, 10 had level II VTT, five had level III VTT, and five had level IV VTT. The present study was approved by the Institutional Ethics Committee of our hospital.

The clinical manifestations of the patients were recorded, including local and systemic symptoms. Before surgery, B‐ultrasound was performed to evaluate the tumor's side, location, diameter, and relationship with renal vessels and collecting system. Enhanced magnetic resonance imaging (MRI) was performed to evaluate the length and invasion of VTT and the presence of BT. Thoracic and abdominal computed tomography (CT) was performed. Hemoglobin, leukocyte count, neutrophil count, lymphocyte count, platelet count, total protein, albumin, serum calcium, blood urea nitrogen, serum creatinine, and alkaline phosphatase were measured before surgery. Blood creatinine was re‐evaluated 1 week after surgery. For patients with clinical symptoms, MRI or bone scan was performed to exclude central nervous system or bone metastasis.

In this study, BT was initially diagnosed by preoperative enhanced CT and/ or enhanced MRI, and then confirmed by postoperative pathological examination. The imaging diagnostic criteria of VTT with BT are shown in Table 1. In the delayed phase of enhanced MRI, BT showed no obvious enhancement, while VTT usually showed enhancement. This was the most important difference between BT and VTT.

**TABLE 1:** The imaging diagnostic criteria of venous tumor thrombus combined with bland thrombus

Item	Content	TT	BT
Enhanced CT	Width of the involved vein	Widened	Widened or normal
	Shape of thrombus	Irregular	Regular
	The edge of thrombus	Rough	Smooth
	Enhanced scanning	Enhanced	Not enhanced; relatively low density
Enhanced MRI	T1WI sequence	Low signal	High signal
	T2WI sequence	Uneven signal	Uniform signal
	DWI sequence	Local or total high signal	Low signal
	Contrast‐enhanced imaging	Uneven enhancement; enhanced degree similar to tumor; neovascularization	No enhancement; low signal

Abbreviations: BT, bland thrombus; CT, computed tomography; MRI, magnetic resonance imaging; TT, tumor thrombus.

### 2.2 Surgical method

For patients with RCC and VTT concomitant with BT, the surgical procedure was determined according to the location and extent of BT (Table 2).

**TABLE 2:** Bland thrombus classification according to the relative position of bland thrombus

BT classification	Definition	Number, n (%)	Preoperative diagram	Postoperative diagram
Group A	Proximal end BT	1 (4.8%)	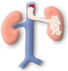	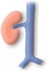
Group B	BT in the contralateral renal vein	2 (9.5%)	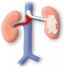	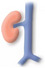
Group C	Distal end BT	12 (57.1%)	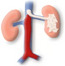	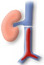
Group D	Multiple BT	6 (28.6%）	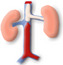 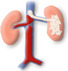 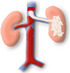	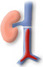

Abbreviation: BT, bland thrombus.

**FIGURE 1:**
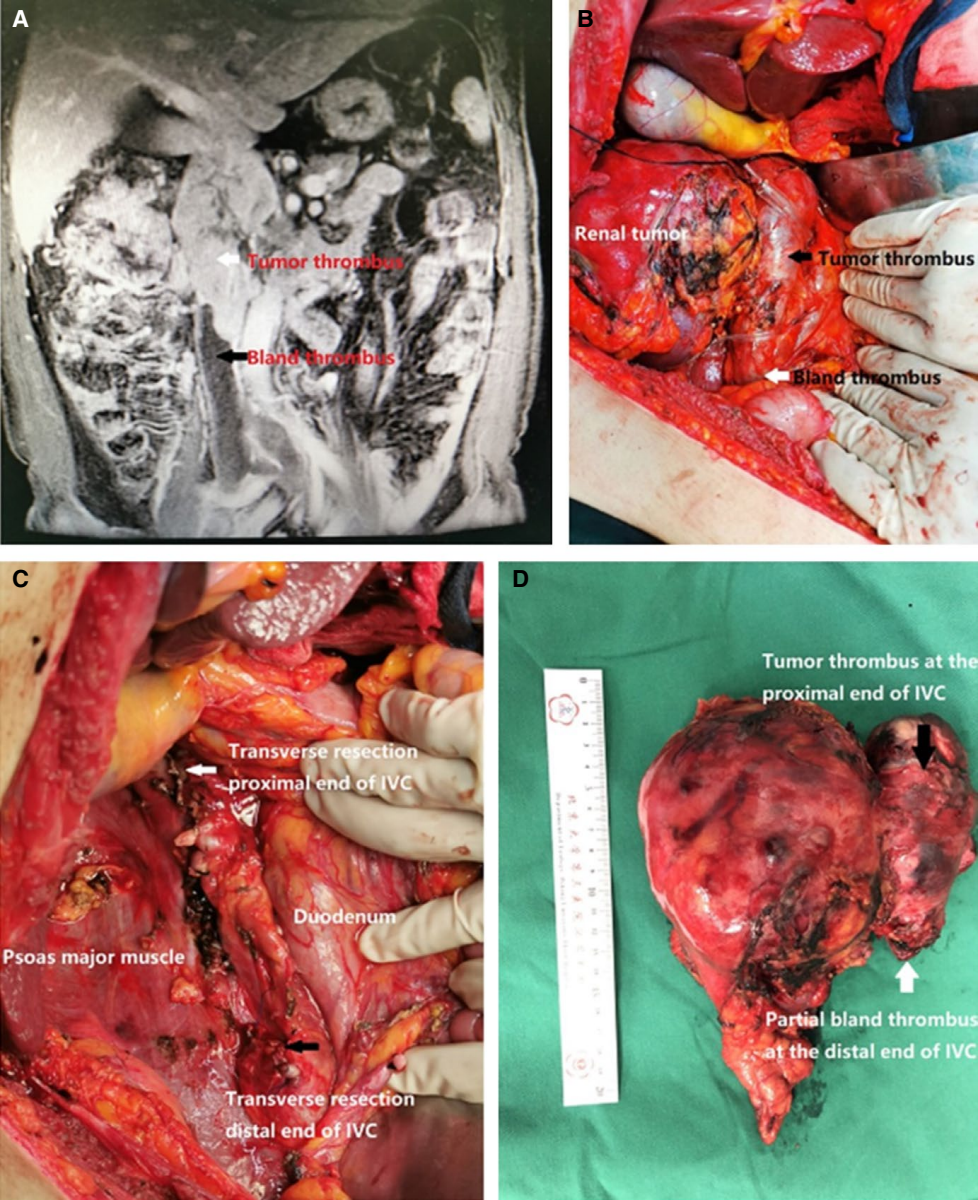
A 58‐year‐old man with right renal cell carcinoma (RCC) with inferior vena cava (IVC) tumor thrombus (TT). (A) Abdominal enhanced magnetic resonance imaging (coronal position) showed the widening of the IVC and TT in the lumen (red arrow). The extent of IVC involvement was about 11.6 cm. Filling defect was seen at the entrance of renal vein into IVC and distal end of the IVC, suggesting the presence of bland thrombosis (BT) (black arrow). (B) Exposure of IVC and related vessels: distal end of IVC, contralateral renal artery or vein, proximal end of IVC. BT in the right renal vein and IVC. The distal end of the IVC and bilateral common iliac and external iliac veins had BTs. (C) We exposed the IVC by dissociating the duodenum to the inside of the abdomen. We transected the distal end of the IVC, contralateral renal vein, and proximal end of the IVC. The right kidney and the TT were removed completely. (D) Postoperative gross specimen showed right renal tumor and IVC TT combined with distal BT

For distal end BT of IVC, if the BT was short, it could be removed with the VTT. If the BT was long, transverse resection of the IVC was required (Figure 1). For patients with IVC wall invasion, we excised the wall of the IVC and the VTT, and sutured the wall of the IVC. The outflow tract of the contralateral renal vein to the IVC was preserved. The distal end of the IVC was incised with scissors and sutured with a 3‐0 vascular suture. BT in the distal end of the transverse incision remained in the IVC.

For proximal end BT of IVC, the top of the BT was accurately determined during the operation, and intraoperative ultrasound was used to assist the diagnosis if necessary. The IVC needed a careful operation to reduce compression as much as possible, so as to avoid displacement of the BT, which may cause pulmonary embolism. The distal end of the IVC, the contralateral renal vein and the proximal end of the IVC were blocked with a vascular blocking band. After the IVC was cut, the VTT and BT were carefully removed to ensure the integrity of the BT.

For BT in the contralateral renal vein, or short BT in the distal end of the IVC, the distal end of the IVC, contralateral renal vein and proximal end of the IVC were blocked with a vascular blocking band during the operation. After the IVC was cut, the VTT and BT were completely removed.

### 2.3 Postoperative complications and follow‐up

Postoperative complications were recorded. The patients were followed up once every 6 months until year 5, and once every year thereafter. The follow‐up included biochemical analysis (renal function), urinary enhanced CT, and chest X‐ray.

### 2.4 Statistical analysis

For comparison of categorical variables, Chi‐square tests were used. For comparison of continuous variables, the Mann‐Whitney *U* test was used. Univariable log‐rank tests and multivariable Cox regressions were conducted to evaluate the prognostic significance of each variable with respect to CSS. Kaplan‐Meier plots were performed to evaluate the influence of BT on CSS. All the statistical analyses were performed using SPSS version 22.0. A *P* value <.05 was considered statistically significant.

## 3 RESULTS

The clinical and pathological data of patients are shown in Table 3. Compared with non‐BT patients, 21 patients (17.1%) with BT had longer operative time (416.76 ± 103.61 vs 314.86 ± 123.00 min, *P* = .001), more surgical blood loss (2738.10 ± 2238.41 vs 1090.98 ± 1395.21 mL, *P* = .004), more red blood cell transfusion (1933.33 ± 2036.99 vs 624.51 ± 926.87 mL, *P* = .009), more plasma transfusion (619.05 ± 831.64 vs 171.57 ± 390.07 mL, *P* = .025), larger maximum width of VTT (32.29 ± 7.01 vs 20.28 ± 8.94 mm, *P* < .001), larger width of VTT at the entrance of the renal vein (26.99 ± 4.47 vs 17.86 ± 6.74 mm, *P* < .001), higher proportion of open surgery (81.0% vs 47.1%, *P* = .006), higher rate of IVC transverse resection (57.1% vs 9.8%, *P* = .001), and higher incidence of postoperative complications (71.4% vs 27.5%, *P* = .011). RCC with VTT and BT increased the difficulty of surgery.

**TABLE 3:** Comparison of clinical and pathologic characters between bland thrombus (BT) patients and non‐BT patients

	Non‐BT (n = 102)	BT patients (n = 21)	*P* value
Gender, n (%)			1.000
Male	76 (74.5%)	16 (76.2%)	
Female	26 (25.5%)	5 (23.8%)	
Age, y, mean ± SD	58.56 ± 10.56	59.00 ± 13.89	.870
BMI, kg/m^2^, mean ± SD	23.56 ± 4.00	23.93 ± 3.68	.696
Side, n (%)			.340
Left	41 (40.2%)	8 (38.1%)	
Right	61 (59.8%)	13 (61.9%)	
ASA grade, n (%)			.335
1	8 (7.8%)	0 (0%)	
2	81 (79.4%)	12 (57.1%)	
3	13 (12.7%)	9 (42.9%)	
Clinical symptoms, n (%)			.126
No clinical symptoms	29 (28.4%)	3 (14.3%)	
Local symptoms	42 (41.2%)	10 (47.7%)	
Systemic symptoms	15 (14.7%)	2 (9.5%)	
Both	16 (15.7%)	6 (28.5%)	
Clinical N stage, n (%)			.336
cN0	48 (47.1%)	7 (33.3%)	
cN1	54 (52.9%)	14 (66.7%)	
Clinical M stage, n (%)			
cM0	70 (68.6%)	14 (66.7%)	.304
cM1	32 (31.4%)	7 (33.3%)	
Mayo classification, n (%)			<.001
0	28 (27.5%)	0 (0%)	
I	34 (33.3%)	1 (4.8%)	
II	24 (23.5%)	10 (47.6%)	
III	9 (8.8%)	5 (23.8%)	
IV	7 (6.9%)	5 (23.8%)	
Hemoglobin, g/L, mean ± SD	122.89 ± 23.76	112.38 ± 19.44	.060
Platelet count, ×10^9^/L, mean ± SD	245.34 ± 100.82	230.00 ± 73.81	.510
Serum calcium, mg/dL, mean ± SD	8.71 ± 0.81	8.55 ± 0.47	.405
Albumin, g/L, mean ± SD	38.29 ± 5.88	38.32 ± 5.67	.982
Alkaline phosphatase, U/L, mean ± SD	93.46 ± 47.35	103.43 ± 60.28	.404
Preoperative serum creatinine, µmol/L, mean ± SD	97.88 ± 56.98	103.71 ± 27.14	.648
Tumor diameter, cm, mean ± SD	8.94 ± 3.50	7.64 ± 2.84	.113
Maximum width of VTT, mm, mean ± SD	20.28 ± 8.94	32.29 ± 7.01	<.001
The width of VTT at the entrance of the renal vein, mm, mean ± SD	17.86 ± 6.74	26.99 ± 4.47	<.001
Surgical approach, n (%)			.006
Laparoscope	54 (52.9%)	4 (19.0%)	
Open	48 (47.1%)	17 (81.0%)	
IVC transverse resection, n (%)			.001
No	92 (90.2%)	9 (42.9%)	
Yes	10 (9.8%)	12 (57.1%)	
Operative time, min, mean ± SD	314.86 ± 123.00	416.76 ± 103.61	.001
Surgical blood loss, mL, mean ± SD	1090.98 ± 1395.21	2738.10 ± 2238.41	.004
Red blood cell transfusion, mL, mean ± SD	624.51 ± 926.87	1933.33 ± 2036.99	.009
Plasma transfusion, mL, mean ± SD	171.57 ± 390.07	619.05 ± 831.64	.025
Pathology type, n (%)			.747
Clear cell RCC	86 (84.3%)	17 (81.0%)	
Non‐clear cell RCC	16 (15.7%)	4 (19.0%)	
Sarcomatoid differentiation, n (%)	18 (17.6%)	4 (19.0%)	1.000
Serum creatinine one week after operation, µmol/L, mean ± SD	113.13 ± 102.98	167.62 ± 203.73	.245
Postoperative complication, n (%)	28 (27.5%)	15 (71.4%)	.011
Postoperative adjuvant targeted therapy, n (%)	60 (58.8%)	11 (52.4%)	.814

There was no significant difference between BT and non‐BT patients in gender, tumor side, age, clinical symptoms, American Society of Anesthesiologists (ASA) grade,^12^ body mass index (BMI), or tumor diameter. The proportion of high‐level VTT (Mayo level II‐IV) in the BT group was 95.2%, compared with 39.2% in the non‐BT group. There was no significant difference in clinical N or M stage, pathological type, pathological grade, or sarcomatoid differentiation.

Twenty‐one patients were classified according to the relative position of BT (Table 2). Group A (proximal end BT of the IVC) had one patient (4.8%), Group B (BT in the contralateral renal vein) had two patients (9.5%), and Group C (distal end BT of the IVC) had 12 patients (57.1%). The average length of BT was 10.39 ± 5.92 cm (range: 1.5‐20 cm). Group D had six patients (28.6%). Four patients had BT in the contralateral renal vein and distal end of the IVC. One patient had BT in the distal and proximal ends of IVC. One patient had BT in the distal and proximal ends of the IVC and contralateral renal vein.

**FIGURE 2:**
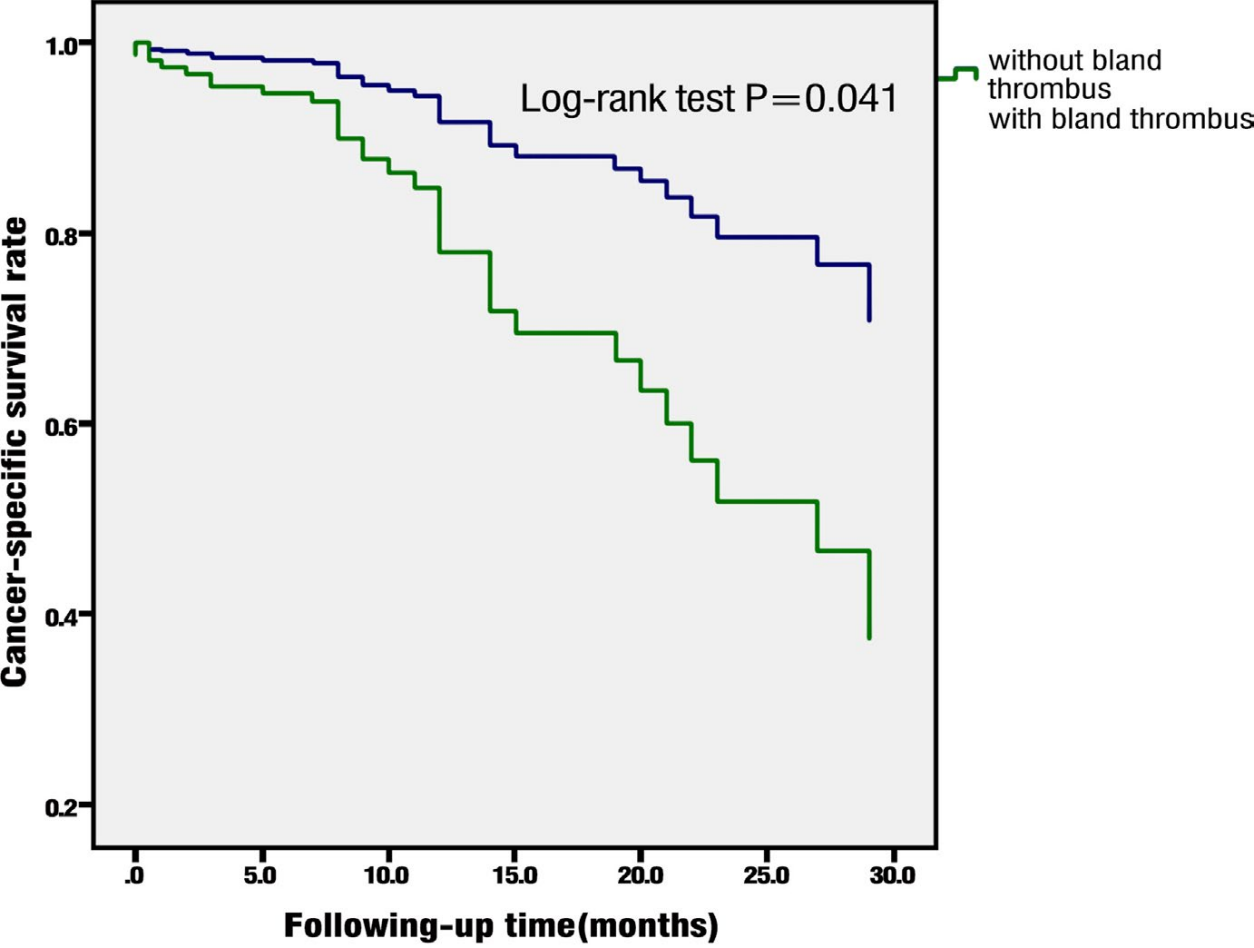
Cancer‐specific survival of bland thrombus (BT) and non‐BT patients

Distant metastasis (hazard ratio [HR] = 3.356, *P* = .004), sarcomatoid differentiation (HR = 6.875, *P* < .001), clear cell RCC (HR = 3.171, *P* = .015), elevated alkaline phosphatase (HR = 2.543, *P* = .029), and BT (HR = 3.323, *P* = .007) were independent risk factors for prognosis (Table 4). The mean CSS of non‐BT patients was 31.7 ± 1.9 months, while that of BT patients was 18.8 ± 1.8 months (*P* = .041) (Figure 2).

**TABLE 4:** Prognosis factors for renal cell carcinoma patients with venous tumor thrombus

Items	Univariable analysis			Multivariable analysis		
	HR	95% CI	*P* value	HR	95% CI	*P* value
M1 stage	3.499	1.726‐7.091	<.001	3.356	1.488‐7.568	.004
Mayo classification	2.207	1.439‐3.385	<.001	—	—	.830
Maximum width of tumor thrombus	1.158	1.085‐1.236	<.001	—	—	.986
Operative approach	2.157	1.023‐4.547	.043	—	—	.884
IVC resection	6.900	2.336‐20.379	<.001	—	—	.361
Operative time	1.007	1.003‐1.011	.001	—	—	.761
Surgical bleeding volume	1.000	1.000‐1.001	.001	1.000	1.000‐1.001	.043
Surgical blood transfusion volume	1.001	1.000‐1.001	.001	—	—	.654
Plasma transfusion volume	1.001	1.000‐1.002	.002	—	—	.915
The width of tumor thrombus at the entrance of the renal vein	1.236	1.109‐1.379	<.001	1.220	1.089‐1.366	.001
Sarcomatoid differentiation	4.039	1.962‐8.314	<.001	6.875	2.907‐16.260	<.001
Clear cell RCC	2.850	1.337‐6.075	.007	3.171	1.257‐7.999	.015
Elevated alkaline phosphatase	3.120	1.474‐6.604	.003	2.543	1.102‐5.869	.029
BT	2.271	1.008‐5.121	.048	3.323	1.378‐8.012	.007

Abbreviations: BT, bland thrombus; CI, confidence interval; HR, hazard ratio; RC, renal cell carcinoma.

## 4 DISCUSSION

Preoperative imaging is important for diagnosis and determination of the extent of BT in patients with RCC with VTT. However, it is easy to misdiagnose or miss BT by imaging. There are similarities between the morphological characteristics of BT and VTT. The main ways to distinguish BT from VTT preoperatively are as follows: (a) Contrast‐enhanced CT: the venous lumen containing VTT becomes wider; the shape of the VTT is irregular, and its edge is mostly rough. After contrast‐enhanced scanning, the lesions in the lumen can be seen to enhancement. BT can also be manifested as widening of the involved venous lumen, or as the normal lumen. Most BTs are regular in shape and smooth edged. After contrast‐enhanced scanning, the lesions in the lumen are generally not enhanced and show low density.^13^, ^14^ (b) Enhanced MRI of the IVC: in T1‐weighted imaging, VTT shows low signal intensity, while BT shows high signal intensity. In T2‐weighted imaging, VTT shows uneven signal intensity, while BT shows even signal intensity. In diffusion‐weighted imaging, VTT shows local or overall signal enhancement, while BT shows decreased signal intensity.^11^, ^15^ VTT shows uneven enhancement and its degree of enhancement is similar to that of the primary sites. Neovascularization shadows can be seen in the arterial phase, and BT usually shows no enhancement and low signal intensity. In the delayed phase of enhanced MRI, BT shows no obvious enhancement, while VTT usually shows enhancement, which is the most important basis for the identification of VTT and BT.

VTT combined with BT might increase the difficulty of surgical treatment.^16^, ^17^ The present study showed that RCC patients with VTT and BT had longer operating time, more surgical blood loss and more blood transfusion. The reasons might be as follows. (a) The proportion of high‐level VTTs in the BT group was higher, accounting for 95.2%, compared with 39.2% in the non‐BT group. The surgical complexity of high‐level VTT is greater than that of low‐level VTT. (b) In BT patients, the IVC usually had complete obstruction, and the rate of IVC transverse resection was higher. In these cases, balloon catheter or milking technique was not used to simplify the operation. (c) If BT was in the distal end of the IVC, it was easy to cause complete obstruction of the IVC. This led to formation of collateral venous circulation, with compensatory veins around the tumor, which caused more surgical blood loss. Therefore, our study suggests that VTT combined with BT might further increase the difficulty of surgery.

There are some specific surgical procedures for patients with BT. For BT in the proximal end of the IVC, renal vein TT in the contralateral side, or short segment TT in the distal end of the IVC, one should carefully remove the VTT and BT after IVC incision, and try to maintain the integrity of the thrombi. For BT in the distal end of the IVC, which has a long range of IVC involvement and cannot be removed during the operation, transverse resection of the IVC is usually required.^18^ The purpose of transverse resection is to prevent BT shedding and causing pulmonary embolism. Some studies have shown that the transverse resection of the IVC might lead to obstruction of IVC reflux, causing lower extremity edema, scrotal edema, and renal insufficiency.^19^ However, in this study, we thought that transverse resection of the IVC was safe and effective, and prevented BT from shedding and causing pulmonary embolism. BT in the distal end of the IVC can cause complete obstruction, and promote formation of collateral venous circulation. When the IVC is completely occluded, the branch veins undergo compensatory dilation, and collateral circulation is usually established. The gonadal vein, adrenal vein, and lumbar vein are the branches of left renal vein; therefore, segmental resection of the IVC has little effect on the left renal function. The right renal vein has fewer and thinner branches. When left renal TT invades the IVC wall, blood can flow from the right renal vein back into the distal end of the IVC after segmental or transverse resection of the IVC. In this circumstance, the collateral circulation channels, such as the lumbar veins, should be preserved as much as possible. If preoperative imaging does not show that the collateral circulation is established, the right renal vein can be reconstructed with an autogenous vein, bovine pericardial patch, or an artificial blood vessel.

The incidence of postoperative complications was 57.1% in patients with VTT and BT, compared with 27.5% in the non‐BT patients. Fifteen BT patients had postoperative complications: one, nine, three, and two patients had Clavien‐Dindo classification grade I, II, IV, and V complications, respectively.^20^ Five patients had serious complications (grade III‐V). Twenty‐eight non‐BT patients had postoperative complications: 4, 21, 1, and 2 patients had grade I, II, III, and IV complications respectively. The average CSS of patients with BT was significantly shorter than that of patients without BT, which suggests that BT is an independent risk factor for poor prognosis.^21^, ^22^, ^23^ Distant metastasis, sarcomatoid differentiation, clear cell RCC, elevated alkaline phosphatase, and BT were the independent risk factors for poor prognosis. Therefore, RCC patients with VTT and BT diagnosed by preoperative imaging need more close follow‐up.

The formation mechanism of BT is still unclear. Some urologists have speculated that RCC is an immunoreactive tumor and may develop an inflammatory, procoagulative surface that triggers formation of BT.^24^ The presence of BT may increase surgical difficulty or extend the operative time. During surgery, BT in the proximal IVC may drop off and cause pulmonary embolism.^25^ It was shown previously that almost half of BT patients required IVC ligation or segmental resection.^5^, ^11^ The presence of BT is related to more obvious clinical symptoms, more surgical blood loss, higher VTT Mayo level, more preoperative metastasis, poorer perioperative outcomes, worse pathological features, and poorer progression‐free survival and overall survival.^5^, ^11^, ^24^ However, no study has explained the mechanism, through which BT affects prognosis. Further studies are needed to explore this question.

There were some limitations to our study. First, it was retrospective and may have been subject to selection bias, and some factors may have affected the outcomes. Second, the surgical procedures were completed by different surgeons in our center, and this may have affected the outcomes of some surgical procedures. Third, the number of patients with BT was small, and classification of BT may not have been completely accurate. The numbers of patients with different classes of BT were small; thus we could not compare the effects of different BT classes on the surgical treatment and prognosis.

## 5 CONCLUSIONS

In the delayed phase of enhanced MRI, BT shows no obvious enhancement, while VTT usually does show enhancement. This is an important difference between BT and VTT on preoperative imaging. The presence of BT increases surgical difficulty, and is correlated with adverse survival outcomes in patients with RCC with VTT.

## CONFLICT OF INTEREST

All the authors made no disclosures.

## AUTHORS’ CONTRIBUTION

ZL, LZ, and PH contributed equally as first authors of this manuscripts. All authors contributed to the design of this project, data collection and analysis, literatures review, making illustration, and contributed to the final manuscript. All authors have read and approved the final submitted manuscript.

## Supporting information


**Figure S1**

**Table S1**

**Data S1**

## Data Availability

The datasets used and/or analysed during the current study are available from the corresponding author on reasonable request.

## References

[1] Ljungberg B , Campbell SC , Cho HY , et al. The epidemiology of renal cell carcinoma. Eur Urol. 2011;60:615‐621. 21741761 10.1016/j.eururo.2011.06.049

[2] Blute ML , Leibovich BC , Lohse CM , Cheville JC , Zincke H . The Mayo Clinic experience with surgical management, complications and outcome for patients with renal cell carcinoma and venous tumour thrombus. BJU Int. 2004;94:33‐41. 15217427 10.1111/j.1464-410X.2004.04897.x

[3] Ljungberg B , Bensalah K , Canfield S , et al. EAU guidelines on renal cell carcinoma: 2014 update. Eur Urol. 2015;67:913‐924. 25616710 10.1016/j.eururo.2015.01.005

[4] Al Otaibi M , Abou Youssif T , Alkhaldi A , et al. Renal cell carcinoma with inferior vena caval extention: impact of tumour extent on surgical outcome. BJU Int. 2009;104:1467‐1470. 19388993 10.1111/j.1464-410X.2009.08575.x

[5] Hutchinson R , Rew C , Chen G , Woldu S , Margulis V . The adverse survival implications of bland thrombus in renal cell carcinoma with venous tumor thrombus. Urology. 2018;115:119‐124. 29499258 10.1016/j.urology.2018.02.019

[6] Quencer KB , Friedman T , Sheth R , Oklu R . Tumor thrombus: incidence, imaging, prognosis and treatment. Cardiovasc Diagn Ther. 2017;7:S165‐S177. 29399520 10.21037/cdt.2017.09.16 PMC5778532

[7] Seles M , Posch F , Pichler GP , Gary T , Pichler M . Blood platelet volume represents a novel prognostic factor in non‐metastatic renal cell carcinoma patients and improves the predictive ability of established prognostic scores. J Urol. 2017;198:1247‐1252. 28716649 10.1016/j.juro.2017.07.036

[8] Wu H , Fan F , Liu Z , et al. The angiogenic responses induced by release of angiogenic proteins from tumor cell‐activated platelets are regulated by distinct molecular pathways. IUBMB Life. 2015;67:626‐633. 26283102 10.1002/iub.1406

[9] Pichler M , Hutterer GC , Chromecki TF , et al. Prognostic value of the Leibovich prognosis score supplemented by vascular invasion for clear cell renal cell carcinoma. J Urol. 2012;187:834‐839. 22245331 10.1016/j.juro.2011.10.155

[10] Li QY , Li N , Huang QB , et al. Contrast‐enhanced ultrasound in detecting wall invasion and differentiating bland from tumor thrombus during robot‐assisted inferior vena cava thrombectomy for renal cell carcinoma. Cancer Imaging. 2019;19:79. 31791422 10.1186/s40644-019-0265-x PMC6889486

[11] Ayyathurai R , Garcia‐Roig M , Gorin MA , et al. Bland thrombus association with tumour thrombus in renal cell carcinoma: analysis of surgical significance and role of inferior vena caval interruption. BJU Int. 2013;110:E449‐E455. 10.1111/j.1464-410X.2012.11128.x 22540981

[12] Davenport DL , Bowe EA , Henderson WG , Khuri SF , Mentzer RM Jr . National Surgical Quality Improvement Program (NSQIP) risk factors can be used to validate American Society of Anesthesiologists Physical Status Classification (ASA PS) levels. Ann Surg. 2006;243:636–644; discussion 641–4. 16632998 10.1097/01.sla.0000216508.95556.cc PMC1570549

[13] Padovan RS , Perkov D , Smiljanic R , Oberman B , Potocki K . Venous spread of renal cell carcinoma: MDCT. Abdom Imaging. 2007;32:530. 16947069 10.1007/s00261-006-9088-x

[14] Lawrentschuk N , Gani J , Riordan R , Esler S , Bolton DM . Multidetector computed tomography vs magnetic resonance imaging for defining the upper limit of tumour thrombus in renal cell carcinoma: a study and review. BJU Int. 2005;96:291‐295. 16042716 10.1111/j.1464-410X.2005.05617.x

[15] Adams LC , Ralla B , Bender YY , et al. Renal cell carcinoma with venous extension: prediction of inferior vena cava wall invasion by MRI. Cancer Imaging. 2018;18:17. 29724245 10.1186/s40644-018-0150-z PMC5934829

[16] Gonzalez J , Gorin MA , Garcia‐Roig M , Ciancio G . Inferior vena cava resection and reconstruction: technical considerations in the surgical management of renal cell carcinoma with tumor thrombus. Urol Oncol. 2014;32:34.e19–34.e26. 10.1016/j.urolonc.2013.01.004 23499500

[17] Blute ML , Boorjian SA , Leibovich BC , Lohse CM , Frank I , Karnes RJ . Results of inferior vena caval interruption by greenfield filter, ligation or resection during radical nephrectomy and tumor thrombectomy. J Urol. 2007;178:440–445. 17561151 10.1016/j.juro.2007.03.121

[18] Pouliot F , Shuch B , Larochelle JC , Pantuck A , Belldegrun AS . Contemporary management of renal tumors with venous tumor thrombus. J Urol 2010; 184: 833‐841; quiz 1235. 20643450 10.1016/j.juro.2010.04.071

[19] Abel EJ , Spiess PE , Margulis V , et al. Cytoreductive nephrectomy for renal cell carcinoma with venous tumor thrombus. J Urol. 2017;198:281‐288. 28268170 10.1016/j.juro.2017.03.011

[20] Dindo D , Demartines N , Clavien PA . Classification of surgical com‐ plications: a new proposal with evaluation in a cohort of 6336 patients and results of a survey. Ann Surg. 2004;240:205‐213. 15273542 10.1097/01.sla.0000133083.54934.ae PMC1360123

[21] Ali ASM , Vasdev N , Shanmuganathan S , et al. The surgical management and prognosis of renal cell cancer with IVC tumor thrombus: 15‐Years of experience using a multi‐specialty approach at a single UK referral center. Urol Oncol. 31, 1298‐1304. 10.1016/j.urolonc.2011.11.001 22169073

[22] Kaushik D , Linder BJ , Thompson RH , et al. The impact of histology on clinicopathologic outcomes for patients with renal cell carcinoma and venous tumor thrombus: a matched cohort analysis. Urology. 2013;82:136‐141. 23642851 10.1016/j.urology.2013.02.034 PMC3710713

[23] Sidana A , Goyal J , Aggarwal P , Verma P , Rodriguez R . Determinants of outcomes after resection of renal cell carcinoma with venous involvement. Int Urol Nephrol. 2012;44:1671‐1679. 23085835 10.1007/s11255-012-0314-x

[24] Smith SA , Travers RJ , Morrissey JH . How it all starts: initiation of the clotting cascade. Crit Rev Biochem Mol Biol. 2015;50:326‐336. 26018600 10.3109/10409238.2015.1050550 PMC4826570

[25] Lee A . VET in patients with cancer – diagnosis, prevention and treatment. Thromb Res. 2008;123:S50‐S54. 18824256 10.1016/j.thromres.2008.08.017

