# Supplementary material for: The influence of venous tumor thrombus combined with bland thrombus on the surgical treatment and prognosis of renal cell carcinoma patients
Source: Cancer Med. 2020 Jul 6;9(16):5860–8. doi: 10.1002/cam4.3264 (PMC7433832; doi:10.1002/cam4.3264)
Supplement: Supplementary file 2 — Table S1 [file CAM4-9-5860-s002.docx]

Supplementary Table 1**.** Comparison of clinical and pathologic characters among different bland thrombus (BT) Groups

|  | Group A | Group B | Group C | Group D |
| --- | --- | --- | --- | --- |
| Patients, n | 1 | 12 | 2 | 6 |
| Sex, n |  |  |  |  |
| Male | 1 | 10 | 1 | 4 |
| Female | 0 | 2 | 1 | 2 |
| Age, yr, mean± SD | 62 | 59.50 ± 10.39 | 62±8.52 | 61.33 ± 6.80 |
| BMI, kg/m^2^, mean ± SD | 23.3 | 25.20 ± 3.66 | 24.06±3.79 | 25.54 ± 4.68 |
| Side, n |  |  |  |  |
| Left | 1 | 6 | 1 | 0 |
| Right | 0 | 6 | 1 | 6 |
| ASA grade, n |  |  |  |  |
| 2 | 0 | 7 | 2 | 3 |
| 3 | 1 | 5 | 0 | 3 |
| Clinical symptoms, n |  |  |  |  |
| No clinical symptoms | 0 | 3 | 0 | 0 |
| Local symptoms | 0 | 4 | 2 | 4 |
| Systemic symptoms | 0 | 1 | 0 | 1 |
| Both | 1 | 4 | 0 | 1 |
| cN stage, n |  |  |  |  |
| cN0 | 0 | 4 | 1 | 2 |
| cN1 | 1 | 8 | 1 | 4 |
| cM stage, n |  |  |  |  |
| cM0 | 0 | 8 | 1 | 5 |
| cM1 | 1 | 4 | 1 | 1 |
| Mayo classification, n |  |  |  |  |
| I | 0 | 0 | 0 | 1 |
| II | 0 | 5 | 2 | 3 |
| III | 0 | 3 | 0 | 2 |
| IV | 1 | 4 | 0 | 0 |
| Hemoglobin, g/L, mean ± SD | 102 | 106.75 ± 15.72 | 106.75±20.27 | 103.83 ± 16.45 |
| Platelet count, ×10^9^/L, mean ± SD | 97 | 219.42 ± 89.14 | 244.75±43.26 | 269.67 ± 71.26 |
| Serum calcium, mg/dl, mean ± SD | 8.5842 | 8.07 ± 1.87 | 8.80±0.36 | 7.64 ± 2.59 |
| Albumin, g/L, mean ± SD | 28 | 36.63 ± 4.15 | 37.77±4.67 | 34.20 ± 8.35 |
| Alkaline phosphatase, U/L, mean ± SD | 88 | 124.42 ± 75.45 | 87±20.57 | 102.33 ± 56.48 |
| Preoperative serum creatinine, µmol/L, mean ± SD | 126 | 108.08 ± 33.85 | 98.5±17.59 | 99.50 ± 14.98 |
| Tumor diameter, cm, mean ± SD | 5.8 | 9.61 ± 4.35 | 5.97±2.83 | 7.29 ± 3.01 |
| Maximum width of VTT, mm, mean ± SD | 21.2 | 36.07 ± 7.76 | 34.2±6.98 | 31.82 ± 7.056 |
| The width of VTT at the entrance of the renal vein, mm, mean ± SD | 17.4 | 25.45 ± 6.85 | 28.27±4.12 | 26.27 ± 5.24 |
| Surgical approach, n |  |  |  |  |
| Laparoscope | 0 | 2 | 2 | 0 |
| Open | 1 | 10 | 0 | 6 |
| IVC transverse resection, n | 0 | 8 | 1 | 3 |
| Operative time, min, mean ± SD | 589 | 434.08 ± 105.26 | 436±55.59 | 387.50 ± 86.60 |
| Surgical blood loss, ml, mean ± SD | 2800 | 3975.00 ± 2159.18 | 2825±2095.03 | 2633.33 ± 1716.59 |
| Red blood cell transfusion, ml, mean ± SD | 3200 | 2766.67 ± 2090.38 | 2000±1665.33 | 2000.00 ± 1314.53 |
| Plasma transfusion, ml, mean ± SD | 1600 | 866.67 ± 866.90 | 800±1131.37 | 700 ± 944.46 |
| Pathology type, n |  |  |  |  |
| Clear cell RCC | 1 | 10 | 1 | 5 |
| Non-clear cell RCC | 0 | 2 | 1 | 1 |
| Sarcomatoid differentiation, n | 0 | 4 | 0 | 0 |
| Serum creatinine one week after operation, µmol/L, mean ± SD | 86 | 166.33 ± 193.71 | 276.75±330.94 | 223.83 ± 269.19 |
| Postoperative complication, n | 1 | 9 | 0 | 5 |
| Postoperative adjuvant targeted therapy, n | 0 | 6 | 2 | 3 |
| BT = bland thrombus; SD = standard deviation; BMI = body mass index; ASA = American Society of Anesthesiologists; VTT = venous tumor thrombus; IVC = inferior vena cava；RCC = renal cell carcinoma. | | | | |
