# Supplementary material for: The influence of venous tumor thrombus combined with bland thrombus on the surgical treatment and prognosis of renal cell carcinoma patients
Source: Cancer Med. 2020 Jul 6;9(16):5860–8. doi: 10.1002/cam4.3264 (PMC7433832; doi:10.1002/cam4.3264)
Supplement: Supplementary file 3 — Data S1 [file CAM4-9-5860-s003.docx]

**Surgical method**

The surgical approach was decided according to the Mayo classification level of VTT. For level 0 VTT, radical nephrectomy was performed. For level I VTT, milking technique was used to squeeze the TT back to the renal vein, or Satinsky forceps were used to partially block the IVC to remove the VTT. For level II VTT, it was necessary to block the distal and proximal ends of the IVC, and the contralateral renal vein (the right renal artery was blocked at the same time for the left renal cancer with TT) to remove the VTT. For level III VTT exceeding the hepatic vein, we disconnected the liver ligament, exposed and dissociated the posthepatic IVC, blocked the IVC under the diaphragm, and dissociated and blocked the first hepatic portal using the pringer method. For level IV VTT, the central tendon of the diaphragm was cut around the IVC, or the diaphragm was cut directly to move the TT from above to below the diaphragm, and the VTT was removed. Cardiopulmonary bypass was established by opening the chest, and the right atrium can be cut under the condition of hypothermic cardiac arrest or non-stop beating.

When the TT invaded the IVC wall and completely blocked the IVC, or the TT invaded more than two-thirds of the circumference of the IVC wall, collateral circulation was fully established, and segmental resection of the IVC was considered. After blocking the distal and proximal ends of the IVC, and healthy renal veins, the veins were incised with scissors, and the corresponding vascular stumps were sutured with 3-0 sutures. When the TT invaded the IVC wall or had long distal BT, the IVC was usually completely obstructed, and the branch veins developed compensatory dilation and collateral circulation was usually established. The left renal vein had gonadal, adrenal, and lumbar vein and other branches, therefore, segmental resection of the IVC had little effect on left renal function. The right renal vein had fewer branches and smaller branches. When the left renal TT invaded the IVC wall, blood flowed from the right renal vein back to the distal end of the IVC after segmental resection of the IVC. In this circumstance, the collateral circulation channels, such as the lumbar vein, were preserved as much as possible. If the collateral circulation was not established, the right renal vein was reconstructed with an autogenous vein, bovine pericardial patch, or artificial blood vessel. BT in the distal end of the transection segment remained in the IVC.
